# Characterizing Swisher Little Cigar–Related Posts on Twitter in 2018: Text Analysis

**DOI:** 10.2196/14398

**Published:** 2019-07-19

**Authors:** Jon-Patrick Allem, Sree Priyanka Uppu, Tess Boley Cruz, Jennifer B Unger

**Affiliations:** 1 Keck School of Medicine of USC Los Angeles, CA United States; 2 Department of Computer Science University of Southern California Los Angeles, CA United States

**Keywords:** little cigar, cigarillo, Swisher, social media, Twitter, tobacco

## Abstract

**Background:**

Little cigars are growing in popularity in the United States, and Swisher is the market leader. The contexts and experiences associated with the use of Swisher-related products is understudied, but such information is available via publicly available posts on Twitter.

**Objective:**

This study aimed to analyze Twitter posts to characterize Twitter users’ recent experiences with Swisher-related products.

**Methods:**

Twitter posts containing the term “swisher” were analyzed from January 1, 2018, to December 31, 2018. Text classifiers were used to identify topics in posts (n=81,333).

**Results:**

The most prevalent topic was *Person Tagging* (mentioning a Twitter account in a post; 32.77%), followed by *Flavors* (eg, Grape and Strawberry; 20.96%) and *Swisher use* (eg, smoke swisher; 17.44%). Additional topics included *Cannabis use* (eg, blunt, roll, and gut swisher; 6.26%), *Appeal* (eg, like Swisher; 5.92%), *Dislike* (eg, posts that showed dissatisfaction with Swisher products; 3.53%), *Purchases* (eg, buy swisher; 1.90%), and *Cigar comparison* (eg, mentions of other cigar products including White-owl and Backwoods; 1.64%).

**Conclusions:**

This paper describes common contexts and experiences associated with the use of Swisher little cigars from the population posting on Twitter in 2018. These online messages may have offline consequences for tobacco-related behaviors, indicating the need for countering from public health officials. Findings should inform us about targets for surveillance, policy, and interventions addressing Swisher little cigars as well as communication planning and tobacco product counter messaging on Twitter.

## Introduction

Little cigars are growing in popularity in the United States, and Swisher is the market leader [1]. Little cigars deliver substantial nicotine doses and relatively more carbon monoxide than cigarettes [2]. Several factors contributing to the growth in little cigar use have been identified, including their availability in flavors, misperceptions about the harms of use, promotional tactics by the tobacco industry, and lower costs [3,4]. For example, adult smokers have suggested that the lower cost of little cigars (compared to cigarettes) was a reason for initiation and continuation of their use [2]. Internal tobacco industry documents have revealed that tobacco companies engaged in a calculated effort to blur the line between little cigars and cigarettes to increase the appeal to cigarette smokers, and the use of flavors facilitated these efforts [5].

The little cigar consumer marketplace, cultural trends, and tobacco product health policies are constantly evolving [6]. The contexts and experiences associated with little cigar use keep changing, making it important to provide timely information on such issues to inform targets for surveillance, policy, and interventions addressing use of little cigars. Public posts on Twitter can be monitored to quickly capture and describe the context of little cigar use in the words of the people who organically discuss this product. In this way, posts to Twitter can serve as a focus group, offering new insights that may be of importance to tobacco control. Among adults in the United States, Twitter is used by 24% of men, 21% of women, 21% of white individuals, 24% of African Americans, and 25% of Hispanics [7]. In this study, we collected data from Twitter to describe Swisher-related conversations in 2018. Our goal was to describe the public’s recent experiences with Swisher, including gaining an understanding of the context in which little cigars are used.

## Methods

### Data Collection

Twitter (twitter.com) posts containing the term “swisher” were obtained from Twitter’s streaming application program interface (filtered stream using the Twitter4J library for collecting tweets without gaps in the collection time) from January 1, 2018, to December 31, 2018. The term “swisher” is in line with prior research on little cigars utilizing data from social media [8]. We recorded a total of 111,263 posts during this period.

### Data Processing

To prepare the data for analyses, we excluded non-English tweets, retweets, instances where Swisher was identified as a surname, and tweets from accounts identified as social bots [9], which resulted in a final analytic sample of 81,333 tweets from 57,838 unique users.

In line with prior research [10,11], all tweets in the analytic sample were subjected to basic normalization (eg, lower case all tweets, removal of extra spaces, punctuation between words, and special characters such as brackets), stop word removal (eg, words such as “a” and “the”), lemmatization (eg, breaking down words into their basic form by removing inflections and variants), normalizing mentions of Twitter accounts (eg, @account_name is replaced by @person, which is a common token for all accounts), removal of nonprintable characters (eg, emoticons or symbols in other languages), and removal of hashtags and URLs. All analyses relied on public anonymized data; adhered to the terms and conditions, terms of use, and privacy policies of Twitter; and obtained institutional review board approval from the authors’ university. To protect privacy, no tweets were reported verbatim in this report.

### Topic Identification

In line with prior research [10,11], tweets were examined using word counts (frequencies), which included a single word and double-word combinations. From this initial assessment, eight dominant topics were identified by the authors, including *Flavors* (eg, Grape and Strawberry), *Swisher use* (eg, smoke swisher), *Purchases* (eg, buy swisher), *Appeal* (eg, like Swisher), *Cannabis use* (eg, blunt, roll, and gut swisher), *Cigar comparison* (eg, mentions of other cigar products including White-owl and Backwoods), *Dislike* (eg, posts that showed dissatisfaction with Swisher products), and *Person Tagging* (mentioning a Twitter account in a post - @person).

Ultimately, each tweet was classified by checking for the presence of any one of the single words or double-word combinations (n-grams). If a tweet consisted of any of the words associated with a topic, the tweet was classified as part of that topic. In sum, we used a rule-based classification script written in Python where each tweet was checked for the existence of a specified set of n-grams representing a topic [10,11]. For each analysis, we present findings in a confusion matrix where the diagonal line indicates the prevalence of a topic and the off-diagonal lines indicate topic overlap. For example, a hypothetical post such as, “Hey! @person Try Swisher’s new grape flavor” could be classified under *Person Tagging* and *Flavors*. The number of posts containing two or more topics would be found at the intersection of the matrix for these topics.

## Results

The eight topics constituted 62.95% of all tweets in the corpus of tweets. The remaining 37.05% of tweets were too varied to be classified into a single topic with meaningful coverage (eg, coverage of each subsequent topic was <1% of total tweets). The most prevalent topic was *Person* *Tagging* (32.77%; see Multimedia Appendix 1 for common phrases found in this topic), followed by *Flavors* (20.96%) and *Swisher use* (17.45%). *Cannabis use* was the next prevalent topic (6.26%), followed by *Appeal* (5.92%), *Dislike* (3.53%), *Purchases* (1.90%), and *Cigar comparison* (1.64%). *Swisher use* and *Flavors* had the most overlap (12.87%), followed by *Flavors* and *Person Tagging* at (5.57%; Textbox 1 and Table 1).

**Topics and common words found in posts along with “Swisher.” These words are meant to provide further context for each topic, are not exhaustive, and are listed in alphabetical order.**

**Person tagging:**

@person

**Flavors:**

- Cherry
- Flavor
- Grape
- Mango
- Peach
- Pineapple
- Pumpkin
- Strawberry

**Swisher use:**

- Hit
- Pass
- Puff
- Smoke
- Try

**Appeal:**

- Crave
- Enjoy
- Like
- Love
- Need
- Want

**Dislike:**

- Damn
- Don’t
- Fuck
- No
- Shit

**Cannabis use:**

- Blunt
- Gut
- Marijuana
- Roll
- Weed

**Purchases:**

- Buy
- Bought
- Grab
- Pay

**Cigar comparison:**

- Backwoods
- White-owl

**Table 1:** Prevalence of topics. The diagonal line indicates the prevalence of the eight topics identified. The off-diagonal lines indicate topic overlap. All values are given as n (%).

	Person tagging	Flavors	Swisher use	Cannabis use	Appeal	Dislike	Purchases	Cigar comparison
Person tagging	26,656 (32.77)	—^a^	—	—	—	—	—	—
Flavors	4533 (5.57)	17,049 (20.96)	—	—	—	—	—	—
Swisher use	3185 (3.92)	10,464 (12.87)	14,182 (17.44)	—	—	—	—	—
Cannabis use	1107 (1.36)	767 (0.94)	1344 (1.65)	5088 (6.26)	—	—	—	—
Appeal	815 (1.00)	855 (1.05)	386 (0.47)	171 (0.21)	4817 (5.92)	—	—	—
Dislike	661 (0.81)	295 (0.36)	287 (0.35)	72 (0.09)	25 (0.03)	2869 (3.53)	—	—
Purchases	310 (0.38)	393 (0.48)	333 (0.41)	134 (0.16)	20 (0.02)	40 (0.05)	1542 (1.90)	—
Cigar comparison	301 (0.37)	296 (0.36)	344 (0.42)	82 (0.10)	34 (0.04)	159 (0.20)	20 (0.02)	1332 (1.64)

^a^Not applicable.

## Discussion

### Principal Findings

The topics identified in this study of Swisher-related posts on Twitter in 2018 provide several insights about the public’s recent experience with little cigars. *Flavors* was a common topic in this study, similar to earlier Twitter-based studies focused on tobacco products like JUUL [10] and hookah [11]. Content analysis of little cigar–related videos posted to YouTube demonstrated that common themes included their candy flavors [12]. Flavors were cited as important reasons for use of little cigars and cigarillos among a national probability sample of US adults in 2014 [3]. Research also suggests that the perception of risk of flavors in little cigars is related to use of these products, specifically, the perception of less risk [13]. Additionally, more than two-fifths of US middle and high school smokers report using flavored little cigars or flavored cigarettes [14]. Taken together, flavors in little cigars may be considered a priority area for federal regulation to reduce appeal of these little cigars to consumers across age groups and to provide uniform restrictions that make it difficult for distributors and consumers to work around local flavor restrictions.

In a previous assessment of public tweets related to little cigars, Stepp and colleagues found that posts often expressed affiliation for specific brands (Swisher Sweets and Black & Mild) as well as reporting smoking activity [15]. In this study, *Purchases, Swisher use, Appeal, Cigar comparison,* and *Person tagging* were common topics in the data. These results together suggest that Twitter users are talking about their smoking activities; comparing and contrasting their preferences for brands; and directly communicating with their followers about such purchases, preferences, and activities. These online messages may have offline consequences on tobacco-related behaviors [16], suggesting that such messages need countering from public health officials.

*Cannabis use* was a common topic in this study. Prior analysis of Instagram posts revealed that Swisher little cigars were often gutted and filled with cannabis [8]. Little cigars may be at the intersection of nicotine and cannabis use, raising major public health concerns, including increased risk of transition between cannabis and tobacco [17], high frequency of use [18], and addiction to tobacco [18]. For example, ever use of marijuana has been shown to be a predictor of initiation of regular little cigar and cigarillo use among US young adults (ages of 18-34 years) [19].

Prior studies rarely reported that Twitter users voiced dissatisfaction with tobacco [10,11]. However, *Dislike* was a topic identified in this study. Although this topic did not strongly overlap with any of the other topics to indicate further context, it suggests that there are general forms of complaints that could be amplified by public health practitioners to discourage tobacco use, in general, or the uptake of little cigar use, in particular.

### Limitations

This study focused on posts on Twitter, and its findings may not generalize to other social media platforms. Data collection relied on Twitter’s streaming application program interface, which prevented collection of tweets from private accounts. Therefore, our findings may not represent the attitudes and behaviors from individuals with private accounts. The posts analyzed in this study were collected from a 12-month period and may not be generalizable to other time periods. Although only one little cigar brand was the focus of this study, Swisher is the little cigar market leader and has been the focus of prior research [8].

### Conclusions

This paper described the common contexts and experiences associated with Twitter discussions about Swisher little cigars in 2018. The predominant conversation topic contained some form of interpersonal communication (person tagging) that would capture the social nature of the posts, flag another person, or refer to them as a notable source. Flavors, the second most common category, are the main characteristic that could be regulated in the future to reduce appeal. Findings should inform targets for surveillance, policy, and interventions addressing little cigars as well as communication planning and tobacco product counter messaging on Twitter.

## Appendix

Multimedia Appendix 1 Word cloud of common phrases found in Person Tagging.
