# Supplementary material for: Characterizing Swisher Little Cigar–Related Posts on Twitter in 2018: Text Analysis
Source: J Med Internet Res. 2019 Jul 19;21(7):e14398. doi: 10.2196/14398 (PMC6676793; doi:10.2196/14398)
Supplement: Multimedia Appendix 1 [file jmir_v21i7e14398_app1.pdf]

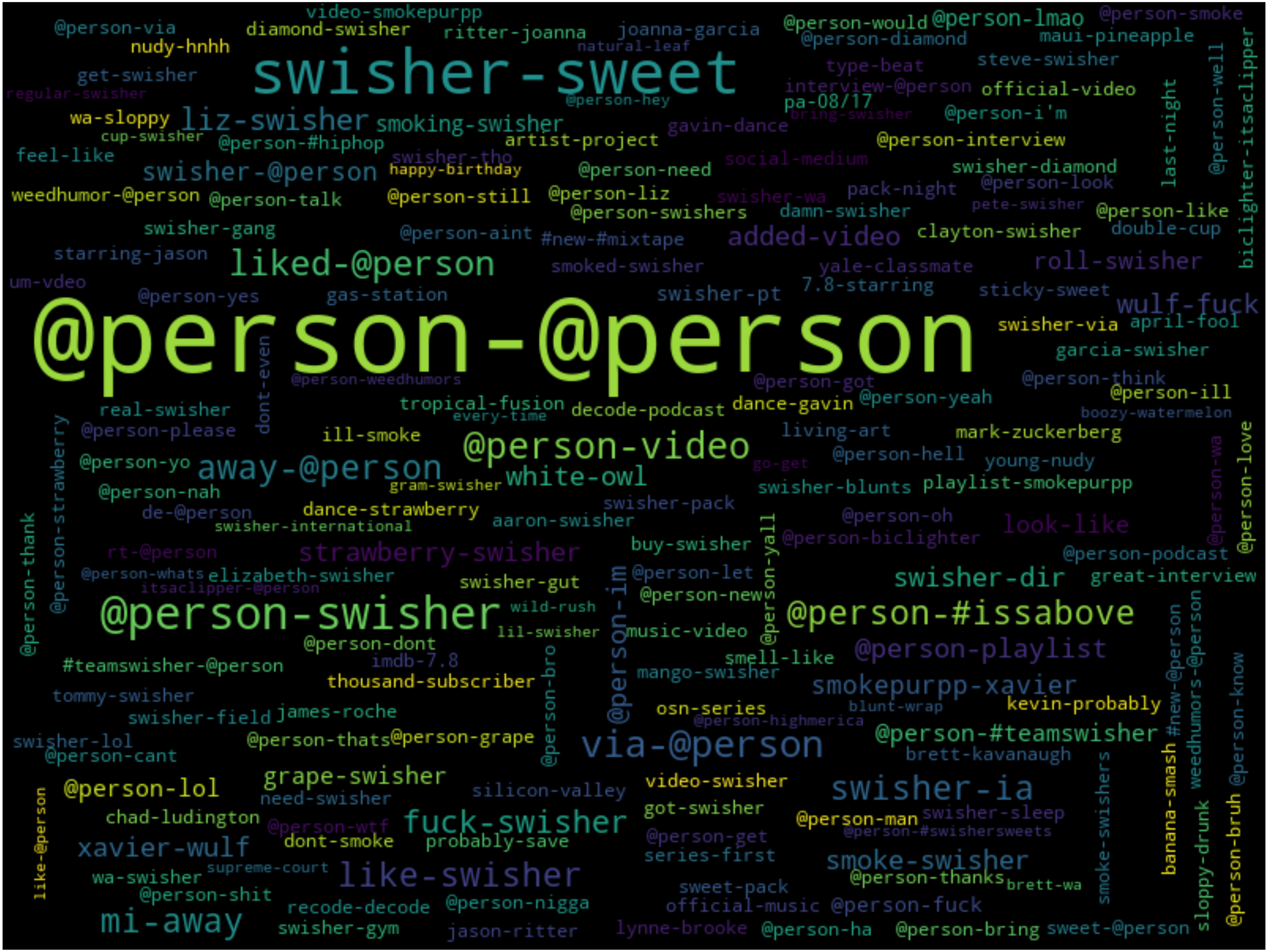

@person-@person
swisher-sweet
@person-swisher
@person-video
via-@person
@person-#issabove
liked-@person
away-@person
swisher-ia
like-swisher
fuck-swisher
mi-away
liz-swisher
swisher-@person
@person-playlist
smokepurpp-xavier
@person-#teamswisher
smoke-swisher
xavier-wulf
added-video
white-owl
strawberry-swisher
swisher-dir
@person-lol
grape-swisher
@person-im
wulf-fuck
roll-swisher
look-like
@person-lmao
smoking-swisher
video-smokepurpp
@person-via
diamond-swisher
ritter-joanna
joanna-garcia
natural-leaf
@person-would
@person-diamond
maui-pineapple
@person-smoke
nudy-hnhh
get-swisher
regular-swisher
type-beat
steve-swisher
interview-@person
official-video
@person-hey
pa-08/17
wa-sloppy
cup-swisher
@person-#hiphop
artist-project
gavin-dance
bring-swisher
@person-i'm
@person-interview
feel-like
swisher-tho
happy-birthday
social-medium
@person-need
swisher-diamond
weedhumor-@person
@person-talk
@person-still
@person-liz
swisher-wa
pack-night
@person-look
@person-swishers
damn-swisher
pete-swisher
@person-like
swisher-gang
@person-aint
#new-#mixtape
clayton-swisher
double-cup
starring-jason
smoked-swisher
yale-classmate
um-vdeo
@person-yes
gas-station
swisher-pt
7.8-starring
sticky-sweet
swisher-via
april-fool
garcia-swisher
@person-weedhumors
dont-even
@person-got
@person-think
@person-ill
real-swisher
tropical-fusion
every-time
decode-podcast
dance-gavin
@person-yeah
boozy-watermelon
@person-please
ill-smoke
living-art
mark-zuckerberg
@person-yo
@person-hell
young-nudy
go-get
gram-swisher
@person-nah
swisher-blunts
playlist-smokepurpp
de-@person
dance-strawberry
swisher-pack
swisher-international
aaron-swisher
@person-oh
@person-thank
@person-strawberry
rt-@person
buy-swisher
@person-yall
@person-biclighter
@person-wa
@person-love
@person-podcast
@person-whats
elizabeth-swisher
itsaclipper-@person
swisher-gut
@person-let
great-interview
@person-new
wild-rush
lil-swisher
music-video
@person-dont
imdb-7.8
smell-like
#teamswisher-@person
thousand-subscriber
mango-swisher
tommy-swisher
osn-series
blunt-wrap
kevin-probably
swisher-field
james-roche
@person-bro
@person-highmerica
#new-@person
weedhumors-@person
@person-know
swisher-lol
@person-thats
@person-grape
brett-kavanaugh
@person-cant
silicon-valley
video-swisher
smoke-swishers
banana-smash
sloppy-drunk
@person-bruh
need-swisher
like-@person
chad-ludington
@person-wtf
got-swisher
@person-man
swisher-sleep
dont-smoke
probably-save
@person-get
@person-#swishersweets
series-first
wa-swisher
supreme-court
@person-thanks
brett-wa
@person-shit
sweet-pack
recode-decode
@person-nigga
official-music
@person-fuck
swisher-gym
jason-ritter
lynne-brooke
@person-ha
@person-bring
sweet-@person
@person-well
biclighter-itsaclipper
last-night
